# Synaptic proteome changes in mouse brain regions upon auditory discrimination learning

**DOI:** 10.1002/pmic.201100669

**Published:** 2012-08-20

**Authors:** Thilo Kähne, Angela Kolodziej, Karl-Heinz Smalla, Elke Eisenschmidt, Utz-Uwe Haus, Robert Weismantel, Siegfried Kropf, Wolfram Wetzel, Frank W. Ohl, Wolfgang Tischmeyer, Michael Naumann, Eckart D. Gundelfinger

**Affiliations:** 1 Institute of Experimental Internal Medicine, Medical School Otto von Guericke UniversityMagdeburg Germany; 2 Leibniz Institute for Neurobiology (LIN)Magdeburg Germany; 3 Institute of Biology Otto von Guericke UniversityMagdeburg Germany; 4 Center for Behavioral Brain Science Research Center of the Otto von Guericke UniversityMagdeburg Germany; 5 Institute of Mathematical Optimization Otto von Guericke UniversityMagdeburg Germany; 6 Institute for Operations Research Department of MathematicsEidgenössische Technische Hochschule Zurich Switzerland; 7 Institute for Biometry und Medical Informatics, Medical School Otto von Guericke UniversityMagdeburg Germany

**Keywords:** Animal proteomics, Auditory learning, Chemical synapse, Isotope-coded protein labelling, Learning and memory, Quantitative mass spectrometry

## Abstract

Changes in synaptic efficacy underlying learning and memory processes are assumed to be associated with alterations of the protein composition of synapses. Here, we performed a quantitative proteomic screen to monitor changes in the synaptic proteome of four brain areas (auditory cortex, frontal cortex, hippocampus striatum) during auditory learning. Mice were trained in a shuttle box GO/NO-GO paradigm to discriminate between rising and falling frequency modulated tones to avoid mild electric foot shock. Control-treated mice received corresponding numbers of either the tones or the foot shocks. Six hours and 24 h later, the composition of a fraction enriched in synaptic cytomatrix-associated proteins was compared to that obtained from naïve mice by quantitative mass spectrometry. In the synaptic protein fraction obtained from trained mice, the average percentage (±SEM) of downregulated proteins (59.9 ± 0.5%) exceeded that of upregulated proteins (23.5 ± 0.8%) in the brain regions studied. This effect was significantly smaller in foot shock (42.7 ± 0.6% down, 40.7 ± 1.0% up) and tone controls (43.9 ± 1.0% down, 39.7 ± 0.9% up). These data suggest that learning processes initially induce removal and/or degradation of proteins from presynaptic and postsynaptic cytoskeletal matrices before these structures can acquire a new, postlearning organisation. In silico analysis points to a general role of insulin-like signalling in this process.

## 1 Introduction

Chemical synapses are pivotal for information transfer and storage within the neuronal circuitry. At the presynaptic site, the protein meshwork of the cytomatrix at the active zone (CAZ) is organised by various multidomain scaffolding proteins. The CAZ is involved in the structural assembly and the functional plasticity of the presynaptic neurotransmitter release apparatus [1,2]. Proteins of the postsynaptic membrane include, among others, neurotransmitter receptors, associated signalling proteins, and cytoskeletal elements, all assembled by a variety of scaffolding proteins into a well-organised structure called the postsynaptic density (PSD) [3,4]. The CAZ and the PSD dynamically change their structure and composition in response to external stimuli and synaptic activity over a time range of seconds to minutes and hours to days. Both pre- and postsynaptic cytomatrices behave biochemically in a similar way and cofractionate into the so-called “PSD” or “synaptic junctional” protein fraction.

New memories consolidate over time from an initially labile state to a more permanent state [5,6]. It is generally accepted that long-term memory formation depends on long-lasting alterations in neurons and, in particular, in the efficacy of their synaptic connections involving structural rearrangements of synapses. These processes are likely to require reprogramming of gene expression at the level of transcription and translation as well as alterations at the posttranslational level, including the modification, localisation, and degradation of proteins and cellular organelles [7,8]. Current views of the role of synaptic plasticity for long-term memory formation have proposed a permissive “unlocking” process with subsequent reassembly of subsynaptic scaffolds [1,3,9,10].

For rodents, the auditory cortex is critical for learning the discrimination of the modulation directions (rising versus falling) of frequency-modulated tones (FMs) in a shuttle box GO/NO-GO procedure [11–13]. Long-term memory formation in this learning paradigm requires glutamate and dopamine receptor activation as well as protein synthesis in the auditory cortex [14–18]. During FM discrimination learning, dopamine critically controls the synthesis of new proteins, which supports the retention of newly acquired memory for longer time periods [15]. In addition, several cortical and subcortical structures connected to the auditory cortex [19] seem to be involved in FM discrimination learning. These include the medial prefrontal cortex as concluded from elevated dopamine responses during and shortly after shuttle box avoidance conditioning to FMs [20], the striatum as the main structure proposed to evaluate the behavioural feedback in reinforcement learning scenarios [21], and the hippocampus, which is known to play a role in the acquisition of active avoidance learning in the shuttle box [22,33]. Consequently, in the present study, the striatum (STR), hippocampus (HIP), frontal cortex (FC), and auditory cortex (AC) of mice were examined for changes in the protein composition of a PSD-enriched synaptic junctional fraction either 6 h or 24 h after FM discrimination training. This fraction includes cytoskeletal and synaptic scaffolding proteins as well as membrane proteins and signalling components that are tightly associated with the synaptic cytomatrices, but is free of light membrane proteins such as synaptic vesicle proteins [24]. At 6 h after learning, we expected to identify proteins required for intermediate-term storage of the newly acquired memory and for its conversion into long-term memory, as suggested by studies on visual and olfactory discrimination learning [25,26]. The later time point was chosen on the basis of our previous studies on FM discrimination, implying that protein synthesis-dependent changes in the auditory cortex support memory retention and subsequent learning for more than 24 h [15,18].

## 2 Materials and methods

### 2.1 Animals

Forty-four male 10 to 16 week-old C57BL/6J mice were used for this study. The animals were housed in groups of four individuals and given free access to standard laboratory chow and tap water on a 12-h light:dark cycle (light on at 6 am). Animal experimentation was approved by the animal care committee of the Land Sachsen-Anhalt in accordance with the regulations of the German Federal Law on the Care and Use of Laboratory Animals and with NIH guidelines.

### 2.2 Experimental strategy

An overview of the experimental strategy is provided in the Supporting Information Fig. S1. Each experimental group comprised four littermates. One of them was trained on the FM discrimination to avoid mild foot-shock in a shuttle box (AV). Two littermates received in the shuttle box either the foot-shocks alone (FS) or the FM tones alone (TS) to control for stress and other non-learning factors during FM discrimination training. The fourth, “naïve” littermate (NV) remained untreated in its home cage during the experimental period. Animals were killed 6 or 24 h after completion of behavioural experiments, and STR, HIP, FC and AC were removed and stored at −80°C. Synaptic junctional (PSD-enriched) protein fractions were prepared from these brain regions, labelled using the ICPL method, and subjected to proteomic analyses. During biochemical and proteomic procedures, the corresponding samples from the littermates of a given experimental group were processed in parallel.

### 2.3 Behavioural experiments

Mice of the AV group were trained in a two-way shuttle box GO/NO-GO task to discriminate between sequences of rising (4–8 kHz, CS+) and falling FMs (8–4 kHz, CS−) to avoid mild foot-shock, as described earlier [11,27]. A training session consisted of 60 trials, i.e. 30 presentations of each CS+ and CS− in pseudo-randomised order, and lasted ≍25 min. The mean (± SEM) intertrial interval was 20 ± 4 s. Control mice received the same number and sequence of either the foot-shock alone (FS) or the tone stimuli alone (TS) as the corresponding trained littermate.

### 2.4 Tissue fractionation

For tissue preparation, animals were killed at indicated time points after training by decapitation. The four brain regions, STR (Bregma 1.54–0.5, excluding tissue from cortex), HIP (according to its easily recognisable structure), FC (Bregma 3.56–1.54; excluding tissue from Bulbus olfactorius), and AC (Bregma −2.06 to −3.4, size: rostrocaudal 2 mm, dorsoventral 1.3 mm; bilateral) were localised on the basis of their stereotaxic coordinates [28], surgically removed (cf. Supporting Information Fig. S1), frozen in liquid nitrogen, and stored at −80°C. Protein fractions enriched for synaptic structures were prepared from the frozen tissues as described [24]. In addition to synaptic cytomatrix proteins and cytoskeletal elements, this fraction contains membrane proteins, like cell adhesion molecules or NMDA receptor subunits, and components of synaptic signalling pathways that are tightly anchored to synaptic scaffolding proteins. Moreover, synaptic junctional protein fractions are known to contain metabolic enzymes that are tightly associated with the synaptic cytoskeleton as well as nucleic acid associated proteins such as RNPs and even histones (for example: [29]]; http://www.synprot.de). The procedure in brief, tissue was homogenised in 300 μL of 10 mM Tris/HCl, pH 8.1, 0.5% Triton X-100 containing protease and phosphatase inhibitors. After incubation for 1 h at 4°C, samples were spun at 100 000 × *g* for 1 h. The resulting pellets were rehomogenised in the same buffer and centrifuged again for 1 h at 100 000 × *g*. The final pellets were resuspended in 500 μL of deionised water and stored at −80°C. SDS-PAGE was performed with aliquots to adjust protein amounts for ICPL labelling.

### 2.5 Proteome analysis

PSD-enriched samples were resuspended in 25 μL 8 M urea with ultrasonication for 1 h at 0°C. Solubilisation was facilitated by addition of 75 μL 50 mM triethylammonium bicarbonate buffer, pH 8.0, containing 0.8% RapiGest (Waters) and 2.5 mM tris(2-carboxyethyl)phosphine. This mixture was shaken at room temperature for 1 h. Reduced cysteine residues were carbamidomethylated by the addition of 10 mM iodoacetamide for 30 min in the dark.

The four corresponding samples of an experimental group (AV, FS, TS, NV) were individually labelled using the ICPL-Quadruplex Kit (Serva, Germany) following the manufacturer's instructions [30]. Aliquots of labelled samples were combined and digested by adding 2.5 μg trypsin (Promega, TrypsinGold) or 10 μg GluC (Roche Diagnostics, sequencing grade) twice for 24 h at room temperature. Digestion efficiency was controlled by SDS-PAGE and silver staining according to [31].

After complete digestion, RapiGest was hydrolysed by addition of TFA to a final concentration of 0.5% and incubation for 1 h at room temperature. Precipitated RapiGest fragments were spun at 16 000 × *g* for 10 min and the resulting supernatant was applied to an Empore universal resin SPE-column (3M, USA), equilibrated with 2 mL methanol and subsequently washed with 0.1% TFA. Resin-bound peptides were washed with 5 mL 0.1% TFA and eluted twice with 0.5 mL 70% ACN, 0.1% TFA. Eluates were pooled and concentrated to a volume of 200 μL in a vacuum evaporator centrifuge (Savant, Thermo). Thereafter, water and pH 3–11 IPG-buffer (GE Healthcare, USA) were added to a final volume of 500 μL. Each sample was used to rehydrate a non-linear 24 cm IPG-strip, pH 3–11 (GE Healthcare) overnight. IEF was performed according to the following regime: max. 50 μA/IPG-strip, 500V /12 h–1000V/3h–2000V/2h–3000V/2h–3000V–5000V/5h–5000V–8000V/5h–8000V/48 h. After completion of peptide focusing IPG-strips were cut into 20 pieces of ≍12 mm length. Peptides were extracted from the IPG-strip pieces by repeated incubation in 25 mM ammonium hydrogencarbonate buffer (pH 8.0) facilitated by ultrasonication at 0°C. Corresponding supernatants were pooled, lyophilised, and further purified prior to MS by nano-scale RP-HPLC (C18-ZipTip, Millipore) following the manufacturer's instructions.

*NanoLC-ESI-iontrap tandem MS*: For MS analysis, samples were redissolved in 10 μL 2% ACN, 0.05% TFA, and subjected to an Ultimate 3000 nano-HPLC (Dionex, Germany). Samples were trapped on a 1 mm PepMap-trapping column for 6 min at 20 μL/min and subsequently subjected to a 75 μm ID, 5 cm PepMap C18-column (Dionex, Germany). Peptide separation was performed by an ACN gradient at 300 nL/min using the following conditions: 0–40 min: 2–50% ACN; 40–50 min: 50–90% ACN; 50–55 min: 90% ACN; 55–70 min: 2% ACN.

Nano-HPLC was coupled online via a nano-spray interface (Bruker, Germany) to an Esquire HCTultra ETDII-Iontrap mass spectrometer (Bruker, Germany). Mass spectra were acquired in positive MS-mode, tuned for tryptic peptides. MS/MS-precursor selection was driven by the recognition of ICPL-quadruplex distribution patterns (see Supporting Information Tables S1 and S2 for detailed MS information). To produce complementary data sets, each selected MS precursor was subjected to both CID- and ETD-driven MS/MS experiments.

CID- and ETD-MS/MS-data sets were processed with DataAnalysis (Bruker Daltonics, Germany), searched with the Mascot algorithm against the Swissprot/UniProt database and subsequently compiled in a ProteinScape SQL-data set (Bruker, Germany). Protein levels were calculated depending on ICPL-labels and expressed relative to the level of the NV littermate of a given experimental group using the WarpLC software package (Bruker, Germany). Proteins differing by more than 10-fold or less than 0.1-fold were excluded from quantitative and qualitative analyses due to technical cut-offs. Detailed experimental information for MS and MS informatics (MSI) are provided in a MIAPE compliant format (Supporting Information Tables S1 and S2).

### 2.6 Immunoblot analysis

For Western blots, PSD-enriched fractions of an additional set of NV and AV (24 h after training) littermates (*n* = 6 per group) were prepared and equal amounts of proteins (adjusted from Coomassie stains) derived from the STR, HIP and FC of individual animals were separated by SDS-PAGE and blotted onto PVDF membranes (Millipore). AC protein recoveries were inadequate for individual Western blot analyses. Quantitative immunoblot analyses were performed for CBL_MOUSE [rabbit anti-CBL (Ab-700, Assay bioTech)] and HRBL_MOUSE [rabbit anti-HRBL (AGFG2) antibody, Assay bioTech]. Primary antibodies were used in a 1:1000 dilution in 2.5% milk/TBS/0.1% Tween-20. Goat anti-rabbit IgG-HRP (SantaCruz) in a dilution of 1:8000 in 2.5% milk/TBS/0.1% Tween-20 was used as secondary antibody. Immunoreactive bands were detected using HRP-chemiluminescence substrate (ECL WesternBlotting Detection Reagent, GE Healthcare) and exposition on Hyperfilm (GE Healthcare). After development films were scanned on calibrated Powerlook 2000 transmitted light scanner (Microtek) and signal quantification was performed by means of QuantityOne Software (Bio-Rad).

### 2.7 Data analysis

The statistical analyses presented here are all at an exploratory level. With the multitude of proteins evaluated in a small number of animals, we did not apply any adjustments for multiple testing in order not to lose too much power. But to exclude purely random positive findings by a summarising consideration, in the FS, TS and AV littermates of an experimental group the relative numbers of proteins with a higher abundance level than in the corresponding NV littermate were determined and compared in a multifactorial repeated measurement ANOVA between treatments, brain areas, and time points. Group means of relative protein levels (FS/NV, TS/NV and AV/NV) were statistically compared against the null hypothesis of no change by a one-sample Student’s *t*-test (hypothesised mean = 1; 3≤*n*≤4 per group). Immunoblot signals were statistically evaluated by Student’s two-tailed *t*-test for unpaired comparisons. Significant difference was assumed at *p* < 0.05.

Network analysis of functional interactions of proteins differentially regulated after aversive learning (AV group) was performed using Ingenuity Pathway Analysis™ (IPA).

## 3 Results

### 3.1 Proteomic analyses

Mice were trained on the FM discrimination to avoid mild foot-shock or, in control groups, received either foot-shock or tone stimuli alone. The proteomes of synaptic junctional cytomatrices (PSD-enriched fractions, [24]) of the brain regions STR, HIP, FC and AC were analysed 6 h and 24 h after training, labelled with isotope-coded tags and compared to corresponding data from naïve mice (the experimental design is illustrated in Supporting Information, Fig. S1). Corresponding samples obtained from the littermates of a given experimental group, comprising one trained animal (AV), one foot-shock control (FS), one tone stimulus control (TS), and one naïve animal (NV), were processed in parallel. For each brain region, more than 2600 different proteins were identified within this crude synaptic protein fraction (Table 1). About one quarter to one third of them provided reliable information for quantification, i.e. ICPL-labelling of at least three different peptides of a given protein. In total, 551 654 peptides, generated by tryptic or GluC-mediated limited digestion of ICPL-labelled protein fractions, were identified.

**Table 1:** Proteomics summary

Step	Number/factor	Samples cumulative
Time points	2	2
Brain areas	4	8
Replicates	4	32
Digests (Tryp/GluC)	2	64
Peptide fractions	20	1280
LC-MS/MS runs/fraction	1	1280

Summary (brain areas)	STR	HIP	FC	AC
Sequenced peptides (peptide scorea) >20)	146 753	137 266	151 638	115 997
Identified proteins (scorea) >80)	4234	3980	4308	3700
Identified proteins (scorea) > 80), non-redundant	2948	2644	2745	2626
Quantifiable proteins non-redundant	693	695	855	881

Summary (total)	
Number of animals	32
Sequenced peptides (peptide scorea) >20)	551 654
Identified proteins (scorea) >80)	16 222
Identified proteins, non-redundant	6789
Quantified proteins	3124
Quantified proteins, non-redundant	1431

a) Score = Mowse score [70].

### 3.2 Regulation of synaptic cytomatrix-associated proteins

In preparations of the AV, FS and TS littermates of a given experimental group, the abundance levels of the quantifiable proteins relative to the corresponding NV values were calculated on the basis of the peak intensities of tryptic or GluC-generated peptides. These relative abundance values were used for the analyses described below. If not otherwise stated, proteins with a similar abundance value as in the NV animal (from factor 0.9 to 1/0.9) were excluded from these analyses.

To test for differences in the protein composition of the PSD-enriched fraction, we first determined in the FS, TS and AV littermates the relative number of proteins with a higher abundance value than in the corresponding NV littermate. Under the null hypothesis of no treatment effect, this value should be near 0.5. ANOVA comparing this relative number between treatment groups, brain regions and time points showed a significant effect of treatment (*p* < 0.001), no significant effect of region (*p* = 0.779) and no significant effect of time (*p* = 0.658). Interestingly, no interactions were evident among the factors treatment, region and time (region × time: *p* = 0.494; treatment × region: *p* = 0.878; treatment × time: *p* = 0.748; treatment × region × time: *p* = 0.362).

The significant treatment effect indicates that the behavioural procedures in the shuttle box induced changes in the protein composition of synaptic mouse brain fractions. To analyse the effects of FM discrimination training and of the control treatments on the protein patterns in more detail, pairwise comparisons (Bonferroni adjusted) were performed. As shown in Table 2, both the FM discrimination training as well as the FS and TS control treatments in the shuttle box induced a significant reduction in the relative numbers of proteins with higher abundance levels than in NV. In the two control groups, the estimated values did not significantly differ from each other and were only slightly smaller than the reference value of 0.5. In contrast, in the AV group, the estimated value was clearly reduced and differed significantly from the numbers monitored in the FS and TS control groups.

**Table 2:** Pairwise comparison of treatments

						95% confidence interval for difference a)	
Treatment		Mean		Mean			
(I)	(J)	(I)	(J)	difference (I−J)	*p*a)	Lower bound	Upper bound
FS	TC	0.481	0.463	0.018	0.084	−0.002	0.038
	AV		0.294	0.187	**0.000**	0.116	0.257
	NV		0.500	−0.019	**0.015**	−0.034	−0.004
TC	FS	0.463	0.481	−0.018	0.084	−0.038	0.002
	AV		0.294	0.169	**0.001**	0.095	0.242
	NV		0.500	−0.037	**0.002**	−0.056	−0.017
AV	FS	0.294	0.481	−0.187	**0.000**	−0.257	−0.116
	TC		0.463	−0.169	**0.001**	−0.242	−0.095
	NV		0.500	−0.206	**0.000**	−0.280	−0.131
NV	FS	0.500	0.481	0.019	**0.015**	0.004	0.034
	TC		0.463	0.037	**0.002**	0.017	0.056
	AV		0.294	0.206	**0.000**	0.131	0.280

Based on estimated marginal means.

a) Repeated measurement ANOVA, adjustment for multiple comparisons: Bonferroni. Significant values (*p* < 0.05) in bold.

**Figure 1:**
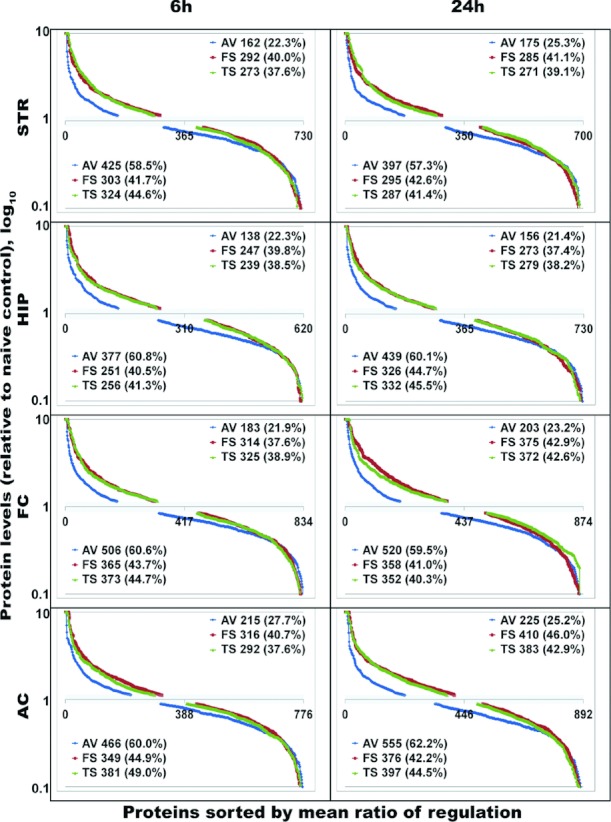
Relative synaptic protein abundance profiles of all experimental groups. Mean values of quantifiable proteins [AV/NV (blue), FS/NV (red) and TS/NV (green)] derived from the striatum (STR), hippocampus (HIP), frontal cortex (FC), and auditory cortex (AC) 6 h or 24 h after behavioural experiments have been sorted according to the size and plotted. Note the prominent downregulation within the trained group (AV) in comparison to the control groups (FS, TS). The numbers and percentage of up and downregulated proteins within the four groups are indicated close to the graphs. Abundance values within a regulation threshold of 10% are excluded from plotting and calculation.

The lack of significant interactions between the factors treatment, region and time implies that global effects dominate over region-specific effects on synaptic protein patterns at 6 h as well as 24 h after training. To further asses the temporal and spatial profiles of synaptic protein changes, the relative protein abundance values were ordered by size within each treatment group and plotted for each brain region and time point separately. As shown in Fig. 1, the patterns of changes observed in the studied brain regions at the two time points after training were indeed very similar. The AV group displayed more downregulated than upregulated proteins, whereas the FS and TS control groups showed nearly balanced numbers of up and downregulated proteins. Overall, the synaptic protein fraction obtained from trained mice showed an average percentage (± SEM) of 59.9 ± 0.5% downregulated proteins and 23.5 ± 0.8% upregulated proteins. In contrast, the control groups showed only marginal discrepancies in the numbers of down and upregulated proteins (FS: 42.7 ± 0.6% downregulated, 40.7 ± 1.0% upregulated; TS: 43.9 ± 1.0% downregulated, 39.7 ± 0.9% upregulated).

To further substantiate this observation, we performed a cluster analysis for all quantifiable proteins. Figure S2 in the Supporting Information shows the heat maps of clusters of the log2 mean values of the relative protein abundance values (FS/NV, TS/NV and AV/NV). Inspection of the heat maps confirms the downregulation of the majority of proteins in the AV groups as compared to FS and TS groups for all brain regions and at both time points analysed.

Brain area-specific correlation plots of proteins 6 and 24 h after the behavioural experiments (Supporting Information Figures S3–S6) were performed to further assess the effects of individual treatments on synaptic protein composition. The plots shown in the lower panels compare the data sets obtained from the controls. The upper four plots compare data sets from AV mice (abscissae) with those from FS or TS controls (ordinates). Here, dots with x≠0, i.e. dots representing proteins with post-training changes in synaptic abundance relative to NV, were predominantly localised in the first and third quadrants. This implies that the synaptic association of these proteins may be regulated during conditioning not only by learning the behavioural meaning (GO or NO-GO) of the two different sounds and the hurdle crossing to avoid the foot-shock, but to some extent also by factors presumably not related to FM discrimination learning, such as the first experience with the experimental stimuli and the training apparatus and stress. On the other hand, dots positioned in quadrants 2 (−/+) or 4 (+/−) as well as dots with x≠0 that are positioned in quadrants 1 (+/+) or 3 (−/−) near the *x*-axis may be particularly indicative of synaptic proteins showing a learning-dependent regulation.

As listed in Table S3 (Supporting Information), statistical analysis of the protein abundance values revealed 40 unique proteins that showed significantly changed abundances in the synaptic fraction after training and/or control treatments relative to the corresponding values in NV mice. Table 3 specifies those 31 proteins that were significantly regulated after FM discrimination training but not after presentation of foot-shock or tones alone. The majority of changes in the synaptic fraction occurred at 24 h posttraining (STR: 10; HIP: 4; FC: 12; AC: 8) whereas at 6 h, the numbers of detected protein changes were smaller (STR: 6; HIP: 1; FC: 6; AC: 6). Assigning these proteins to functional categories [29] revealed that the majority of them (18 out of 31) relate to cell structure components, i.e. the cytoskeleton, scaffolds, and extracellular matrix. The remaining proteins are potentially involved in the regulation of transcription and translation (9), endocytosis (2) and posttranslational modification (2). Strikingly, all of these proteins showed a reduced relative abundance in the synaptic fraction after the learning experience, without an accompanying significant upregulation of other proteins. As the input amounts were normalised for all samples, these findings suggest that a training-induced relative downregulation of a set of synaptic proteins was accompanied by a non-significant upward shift in the relative abundance of all the remaining proteins.

**Table 3:** Avoidance conditioning-induced regulation of protein abundances in synaptic cytomatrices

Swissprot ID	Gene description	Brain region	Time point	Regulation*		
				AV/NV	FS/NV	TS/NV
**Cytoskeleton and scaffolding proteins**						
BSN_MOUSE	Protein bassoon	STR	6 h	0.52*	0.57	0.72
DYH5_MOUSE	Dynein heavy chain 5, axonemal	FC	24 h	0.66*	1.08	1.02
DYH8_MOUSE	Dynein heavy chain 8, axonemal	STR	24 h	0.64*	0.89	0.89
MACF1_MOUSE	Microtubule-actin cross-linking factor 1	HIP	24 h	0.55*	1.18	0.74
MAP1A_MOUSE	Microtubule-associated protein 1A	FC	24 h	0.48*	1.06	0.84
MY18A_MOUSE	Myosin-XVIIIa	AC	24 h	0.57*	1.01	1.45
MYO5A_MOUSE	Myosin-Va	AC	6 h	0.65*	1.55	1.12
MYO5A_MOUSE	Myosin-Va	AC	24 h	0.49*	0.95	0.47
NFH_MOUSE	Neurofilament heavy polypeptide	AC	24 h	0.46*	1.47	1.28
NRAP_MOUSE	Nebulin-related-anchoring protein	FC	24 h	0.64*	1.32	1.10
OBSCN_MOUSE	Obscurin	HIP	6 h	0.54*	1.42	1.13
PCLO_MOUSE	Protein piccolo	FC	24 h	0.49*	0.77	0.77
PCLO_MOUSE	Protein piccolo	STR	24 h	0.50*	0.86	0.79
PLEC1_MOUSE	Plectin-1	FC	6 h	0.75*	1.01	0.75
SPTA2_MOUSE	Spectrin alpha chain, brain	FC	6 h	0.68*	1.03	1.12
SPTB2_MOUSE	Spectrin beta chain, brain 1	AC	24 h	0.59*	0.88	0.70
SPTB2_MOUSE	Spectrin beta chain, brain 1	FC	24 h	0.59*	1.45	1.56
SPTB2_MOUSE	Spectrin beta chain, brain 1	STR	24 h	0.48*	0.79	0.66
TITIN_MOUSE	Titin	AC	6 h	0.62*	0.90	0.98
TITIN_MOUSE	Titin	AC	24 h	0.54*	1.03	0.94
TITIN_MOUSE	Titin	FC	6 h	0.61*	1.09*	0.85
TITIN_MOUSE	Titin	FC	24 h	0.57*	0.98	0.88
TITIN_MOUSE	Titin	HIP	24 h	0.67*	1.04	0.97
TITIN_MOUSE	Titin	STR	6 h	0.67*	1.06	1.10
TITIN_MOUSE	Titin	STR	24 h	0.60*	0.93	0.83
TPPP_MOUSE	Tubulin polymerisation-promoting protein	AC	6 h	0.44*	1.36	0.83
**Extracellular matrix**						
CO4A4_MOUSE	Collagen alpha-4(IV) chain	FC	24 h	0.75*	1.00	0.86
COBA2_MOUSE	Collagen alpha-2(XI) chain	HIP	24 h	0.46*	0.81	0.71
COGA1_MOUSE	Collagen alpha-1(XVI) chain	FC	6 h	0.63*	0.81	0.60
**Trafficking/endocytosis**						
HRBL_MOUSE	HIV-1 Rev-binding protein-like protein	AC	24 h	0.60*	0.97	0.95
HRBL_MOUSE	HIV-1 Rev-binding protein-like protein	FC	6 h	0.64*	0.87	0.89
HRBL_MOUSE	HIV-1 Rev-binding protein-like protein	HIP	24 h	0.67*	0.91	0.78
HRBL_MOUSE	HIV-1 Rev-binding protein-like protein	STR	24 h	0.79*	0.99	0.88
U119B_MOUSE	Protein unc-119 homolog B	STR	6 h	0.66*	0.94	0.94
U119B_MOUSE	Protein unc-119 homolog B	STR	24 h	0.59*	0.62	0.73
**Ubiquitination**						
CBL_MOUSE	E3 ubiquitin-protein ligase CBL	AC	6 h	0.73*	1.00	1.04
CBL_MOUSE	E3 ubiquitin-protein ligase CBL	AC	24 h	0.64*	0.98	0.96
CBL_MOUSE	E3 ubiquitin-protein ligase CBL	FC	24 h	0.64*	1.08	0.92
CBL_MOUSE	E3 ubiquitin-protein ligase CBL	STR	6 h	0.70*	0.87	0.97
**DNA and RNA binding/transcription/translation**						
GAS8_MOUSE	Growth arrest-specific protein 8	FC	24 h	0.68*	0.95	0.86
H2AW_MOUSE	Core histone macro-H2A.2	STR	24 h	0.49*	0.58	1.32
H4_MOUSE	Histone H4	AC	6 h	0.64*	1.02	0.85
H4_MOUSE	Histone H4	AC	24 h	0.67*	0.87	0.82
H4_MOUSE	Histone H4	FC	24 h	0.51*	0.97	0.99
H4_MOUSE	Histone H4	STR	24 h	0.52*	1.00	0.81
HNRPM_MOUSE	Heterogeneous nuclear ribonucleoprotein M	FC	6 h	0.71*	1.28	1.10
HNRPM_MOUSE	Heterogeneous nuclear ribonucleoprotein M	STR	6 h	0.45*	0.78	0.70
LSD1_MOUSE	Lysine-specific histone demethylase 1	STR	24 h	0.44*	0.66	0.61
NUCL_MOUSE	Nucleolin	STR	24 h	0.47*	0.64	0.70
PIN4_MOUSE	Peptidyl-prolyl cis-trans isomerase	STR	6 h	0.73*	0.94	1.05
SLTM_MOUSE	SAFB-like transcription modulator	FC	24 h	0.67*	0.82	0.85
SRP68_MOUSE	Signal recognition particle 68 kDa protein	AC	6 h	0.67*	0.91	0.95
**Kinases**						
ROCK2_MOUSE	Rho-associated protein kinase 2	FC	24 h	0.35*	0.47	0.74

a) Mean protein abundances in PSD-enriched fractions derived from trained mice (AV), foot-shock controls (FS), and tone controls (TS) normalised to the corresponding values from naïve mice (NV).

* *p* < 0.05, significantly different from 1 (one sample *t*-test; 3≤*n*≤4 per group).

**Figure 2:**
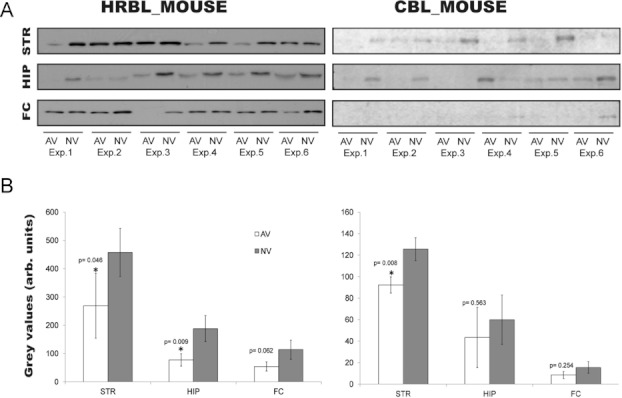
Quantitative immunoblot analysis for HRBL and CBL in trained versus naïve animals. (A) Comparative Western blots of PSD-enriched fractions from striatum (STR), hippocampus (HIP) and frontal cortex (FC) from aversively trained (AV) and naïve (NV) mice probed with antibodies against the ArfGAP HRBL and the E3 ubiquitin ligase CBL. (B) Quantification of the data in panel A. In three cases, the observed downregulation is significant (Student’s *t*-test).

To confirm observed regulations, we have paradigmatically assessed the regulation of the E3 ubiquitin ligase CBL and of HRBL, an ArfGAP protein potentially associated with the Eps15-homology domain (EH) network, by FM discrimination training. PSD-enriched protein fractions of STR, HIP and FC were prepared from six mice 24 h after training (AV) and from six naive siblings (NV) and subjected to immunoblot analyses (Fig. 2). A significant downregulation upon training was observed for HRBL in STR and HIP and for CBL in STR. In the other examples also a downregulation was observed that was, however, not significant.

## 4 Discussion

The findings of the present study suggest that training of mice to discriminate the modulation direction of FM tones in order to avoid mild foot-shock reduces the association of proteins with synaptic cytomatrices derived from striatal, hippocampal, and cortical brain regions 6 h and 24 h after training when compared to naïve, untrained mice. Such a reduction was significantly smaller in control mice that received either the FM tones or the foot-shocks alone, suggesting that the observed molecular changes could relate to FM discrimination learning and memory.

Memory-supporting synaptic plasticity phenomena are assumed to depend on the combined action of different mechanisms, including processes that create the potential for a lasting change in synaptic efficacy and processes responsible for the expression of plasticity [10]. Both permissive and stabilising processes may require local translation of mRNAs downstream of mammalian target of rapamycin (mTOR) pathways. Consistent with this view, our previous studies on FM discrimination in rodents have suggested that translation-dependent changes with different kinetics and behavioural consequences are induced in the brain during learning [14,15,18]. Whereas one process enables storage and retrievability of the newly acquired memory until the next training session performed 1 day later, another process, downstream of D1-like dopamine receptors and mTOR, enhances the predisposition of relevant neural networks for learning and memory formation on subsequent training days. Both processes might involve adaptive changes of the synaptic proteome.

PSD proteins show a surprisingly high rate of turnover or mobility, including “structural” proteins, e.g. actin, and the PSD-95 family of scaffolds [3,4,32]. They are dynamically regulated by protein modifications, such as phosphorylation or ubiquitination, which may decide about dynamic redistribution, proteasome-dependent degradation or local synthesis of specific proteins. Thus abundance of a protein in the biochemical synaptic junctional protein fraction may depend on either its degradation and synthesis rates during plasticity processes or on the strengths of protein–protein interactions that can be regulated by posttranslational modification. Accordingly, in the present study, the reduced abundance of proteins in the PSD-enriched fraction derived from trained mice might reflect, in part, learning-induced processes that reduce the association of proteins with subsynaptic cytomatrices. This is consistent with the critical importance of a majority of the regulated proteins for the (re-)organisation of components of the cellular structure, i.e. the cytoskeleton, scaffolds, and the extracellular matrix. Plectin and MACF1, for example, belong to a family of large multidomain molecules that have various functions to link cytoskeletal elements together and to regulate their dynamics [33–35]. They show widespread occurrence in neural cells and tissues, where they may also act as scaffolding platforms for proteins involved in signalling. Similarly, the giant protein titin is well known as a scaffold protein aiding myofibrillar assembly [36]. Although titin is also expressed in cells other than myocytes, its expression in neural cells has not been described in detail to date. Recent evidence has established a role for titin as a regulatory node integrating diverse signalling pathways in cardiomyocytes. These include, beyond the known mechanical functions of the molecule, phosphorylation of the titin springs, integration of various signalling pathways, the participation in hypertrophic gene regulation, and protein quality control (reviewed in [36]). For obscurin–an interaction partner of titin in myocytes that has been considered to promote cell-matrix contacts at the neuromuscular junction–a recent study revealed important functions in vertebrate brain development [37]. Moreover, the nebulin-related protein NRAP, another protein related to the motile cytoskeleton of the muscle [38], is found to be downregulated in the AV group. This suggests that these proteins may play a yet unknown role in synaptic (or neural) plasticity processes. With bassoon and piccolo, two giant components of the presynaptic cytoskeletal matrix are found to be downregulated. Consistently, a strong regulation of CAZ components during processes of homeostatic synaptic plasticity has been observed [39].

The reduced occurrence of such proteins in the synaptic fraction of trained mice implies that the subsynaptic cytoskeleton and its connection to the basic scaffold of the PSD or the presynaptic cytomatrix are potential targets for learning-induced modulation. This view is supported by the demonstration of a downregulated expression of cytoskeletal proteins 4 h following the induction of long-term potentiation [40], a well-characterised cellular form of synaptic plasticity thought to underlie learning and memory. Moreover, a recent study on the global proteome of the rat hippocampal dentate gyrus after spatial learning revealed an early increase in proteasome-mediated protein degradation and a dynamic regulation of variants of actin, neurofilament, and tubulin throughout the 24-hour posttraining period under consideration [41]. This implies that learning-induced processes may modulate the monomer/polymer balance of major components of the cytoskeleton for up to at least 24 h. In the light of these considerations, the present results may be indicative for learning-induced actions on the cytoskeleton that expand the scaffolds that hold together the molecular protein machines at the synapse thus enabling plastic reorganisation.

An evaluation of the relative numbers of proteins with synaptic abundance changes implies global rather than region- and/or time-specific effects of behavioural treatments (Table 2). Ingenuity Pathway Analysis (IPA) including those 31 proteins that were significantly regulated after FM discrimination training irrespective of region and time suggests that molecular pathways of the insulin-like growth factor (IGF)-related system (comprising the ligands insulin, IGF-1 and IGF-2 [42]) may be involved in determining the training-induced effects on the protein composition of the synaptic fraction (Supporting information S7–S9). Region-specific IPA confirmed this finding for STR, FC and AC. In HIP, the number of only 5 significantly training regulated proteins might be too small for a compelling IPA. Region- and time-specific IPA provides evidence for an involvement of the IGF-related system in STR and FC at 24 h and in AC at 6 and 24 h posttraining. The lack of evidence for an IGF-related system involvement in STR and FC at 6 h after training might reflect differences in the kinetics of activation of the diverse system components. This is consistent with studies on rats demonstrating that insulin/insulin receptor signalling is activated already at 1 h whereas IGF-2 signalling is not induced before 9 h after behavioural training, with a temporal overlap of increased activities of both components at 24 h [43,44].

Like the dopamine system, the IGF-related system is a major component of neural reward circuits; both systems may operate in concert and recruit converging intracellular pathways [45–47]. The IGF-related system was shown to modulate mechanisms involved, among others, in cytoskeleton and scaffold reorganisation, neuronal excitability, dendritic spine and synapse formation, mTOR-dependent synaptic plasticity and memory formation [48–52]. CBL proteins are E3 ubiquitin ligases that may interact with the insulin receptor and negatively regulate receptor tyrosine kinases, such as the IGF-1 receptor, via endocytic mechanisms [47,53,54]. CBL-null mice exhibit a marked improvement of insulin action [55], normal spatial learning, but enhanced long-term memory and synaptic plasticity [56]. This is associated with enhanced paired-pulse facilitation at glutamatergic hippocampal synapses, suggesting that presynaptic functions related to neurotransmitter release are involved. Thus, CBL appears to work in opposition to insulin and to memory formation. This in turn implies that a downregulation of CBL in relevant brain regions, as observed in the present study after training, might be supportive for learning and memory. The isoform composition and phosphorylation of titin are also subject to regulation by insulin signalling, as shown in cardiomyocytes [57]. However, in neural cells and tissues, the regulation of expression and function of titin are far from being clear. Another training-regulated protein, HRBL (HIV-1 Rev-binding protein-like protein), is a component of the EH network of the eukaryotic cell. The EH network is supposed to have principal functions in cytoskeleton reorganisation and in the regulation of endocytosis, including growth factor- and insulin-dependent mechanisms [58]. Endocytic mechanisms have recently been implicated in IGF-2-mediated memory enhancement, presumably via restructuring of synapses and/or homeostatic synaptic scaling, which may enhance the system capacity [42].

Learning an olfactory discrimination task has been shown to induce an enhancement of learning capability accompanied by a series of physiological and morphological modifications in piriform cortex pyramidal neurons, which occur and then disappear at different times over a posttraining period of about 1 week (for review, see [59,60]). In particular, intrinsic neuronal excitability is increased, synaptic transmission is enhanced, and the number of dendritic spines is increased. Learning-induced modification of neuronal excitability was also shown following classical conditioning and spatial learning, in hippocampal, cortical and cerebellar neurons, and in *Hermissenda*, suggesting that it is a cellular mechanism of learning that is conserved across species and tasks. Such long-lasting activity-dependent changes may require neuromodulatory actions such as insulin and D1-like dopamine receptor activity, depend on protein synthesis and cytoskeletal reorganisation, enhance the predisposition for induction of long-term potentiation, and correlate with learning abilities [51,52,61–65]. As such learning-related modifications are spread throughout the neuronal ensemble, and as they disappear without memory loss, they probably are not mechanisms by which specific memories are stored. Rather, they may enable relevant neuronal ensembles to enter a “learning mode” for a time window in which activity-dependent synaptic modifications are more likely to occur [60].

In accordance with these findings, cortical map plasticity improves auditory discrimination learning–presumably by increasing the number of responsive circuits in multiple brain regions–and subsides even though behavioural performance remains stable [66]. Assuming similar long-lasting adaptations also to occur after FM discrimination learning would be a plausible explanation of the present findings. The training-induced regulation of members of the spectrin, myosin and microtubule-associated protein families, which have previously been implicated in the modulation of network excitability, synaptic plasticity and memory [67–69], would speak in favour of this hypothesis. To address this question further and, more specifically, to distinguish between synaptic proteome changes enabling long-term memory storage and those enhancing the predisposition of relevant networks for subsequent learning, future studies will be required, e.g. after overtraining and after training suspension.

In conclusion, the present study on long-term processes of auditory discrimination memory in mice revealed complex synaptic proteome changes in cortical and limbic brain regions during memory consolidation. These changes involve primarily cytoskeletal and synaptic scaffolding proteins, suggesting that learning may loosen the association of proteins with synaptic cytomatrices to facilitate subsequent long-lasting plastic rearrangements.

## Abbreviations

AC: auditory cortex
AV: avoidance learning group
CAZ: cytomatrix at the active zone
CS: conditioned stimulus
EH: Eps15 homology
ETD: electron transfer induced dissociation
FC: frontal cortex
FM: frequency-modulated tone
FS: foot-shock stimulated control group
HIP: hippocampus
ICPL: isotope coded protein labelling
IPA: Ingenuity Pathway Analysis
NV: naïve untreated group
PSD: postsynaptic density
STR: striatum
TS: tone stimulated control group

## Notes

Figure S1 Experimental strategy. (A) Workflow. Mice were trained in a shuttle box to discriminate between rising and falling FM tones. Six or 24 h after avoidance training, mice were sacrificed and striatum (STR), hippocampus (HIP), frontal cortex (FC), and auditory cortex (AC) were removed. Synaptic proteins (PSD-enriched fraction) were collected for each brain region and animal separately. Proteins were labelled by means of ICPL-quadruplex labelling technology and digested with trypsin or GluC endopeptidases. Resulting peptide mixtures were separated by IEF followed by nano-reversed phase HPLC. MS/MS-data sets of four independent replicates were compiled in a database, searched with the Mascot algorithm, and protein abundances relative to the corresponding data from NV mice were calculated. (B) Experimental design of the study. Four male mice (littermates) were either trained or subjected to one of the control treatments and killed after 6 h or 24 h.

Figure S2 Cluster analysis was performed using the mathematical software package "DanteR" (Pacific Northwest National Laboratory/http://omics.pnl.gov). Log_2_ of mean values of relative protein levels (FS/NV, TS/NV and AV/NV) were clustered using the K-means algorithm (K=15) on Euclidean distance metrics without data scaling. Protein accession numbers (Swissprot/Uniprot) are printed on right side of each cluster map. They are readable after zooming in the online publication (http://www.SynProt.de/Auditory_discrimination_learning/). In contrast to most other analyses, where proteins with a similar abundance value as in the NV animal (from factor 0.9 to 1/0.9) were excluded, cluster analysis included all identified quantifiable proteins.

Figure S3 Correlation plots of relative synaptic levels of striatal proteins. Mean abundances relative to NV of striatal proteins monitored 6 h (left) and 24 h (right) after behavioural experiments are plotted on a double logarithmic scale, comparing AV and FS (upper part), AV and TS (middle part), and FS and TS (lower part). Each data point represents a unique protein. Spot colours other than gray correspond to those used in Table S3 (Supporting Information). The percentage of proteins present in each quadrant is given. Proteins with abundance values similar to the NV group (0.9 - 1/0.9) are excluded from plotting and calculation. Corresponding Swissprot/UniProt database accession numbers are available in the interactive plots, which will be available on http://www.synprot.de/Auditory_discrimination_learning/).

Figure S4 Correlation plots of relative synaptic levels of hippocampal proteins. Mean abundances relative to NV of auditory cortex proteins monitored 6 h (left) and 24 h (right) after behavioural experiments are plotted on a double logarithmic scale, comparing AV and FS (upper part), AV and TS (middle part), and FS and TS (lower part). Each data point represents a unique protein. Spot colours other than gray correspond to those used in Table S3 (Supporting Information). The percentage of proteins present in each quadrant is given. Proteins with abundance values similar to the NV group (0.9 - 1/0.9) are excluded from plotting and calculation. Corresponding Swissprot/UniProt database accession numbers are available in the interactive plots, which will be available on http://www.synprot.de/Auditory_discrimination_learning/).

Figure S5 Correlation plots of relative synaptic levels of frontal cortex proteins. Mean abundances relative to NV of frontal cortex proteins monitored 6 h (left) and 24 h (right) after behavioural experiments are plotted on a double logarithmic scale, comparing AV and FS (upper part), AV and TS (middle part), and FS and TS (lower part). Each data point represents a unique protein. Spot colours other than gray correspond to those used in Table S3 (Supporting Information). The percentage of proteins present in each quadrant is given. Proteins with abundance values similar to the NV group (0.9 - 1/0.9) are excluded from plotting and calculation. Corresponding Swissprot/UniProt database accession numbers are available in the interactive plots, which will be available on http://www.synprot.de/Auditory_discrimination_learning/).

Figure S6 Correlation plots of relative synaptic levels of auditory cortex proteins. Mean abundances relative to NV of auditory cortex proteins monitored 6 h (left) and 24 h (right) after behavioural experiments are plotted on a double logarithmic scale, comparing AV and FS (upper part), AV and TS (middle part), and FS and TS (lower part). Each data point represents a unique protein. Spot colours other than gray correspond to those used in Table S3 (Supporting Information). The percentage of proteins present in each quadrant is given. Proteins with abundance values similar to the NV group (0.9 - 1/0.9) are excluded from plotting and calculation. Corresponding Swissprot/UniProt database accession numbers are available in the interactive plots, which will be available on http://www.synprot.de/Auditory_discrimination_learning/).

Figure S7 Analysis of regulated protein networks after aversive learning-summary of all brain areas and time points. Network analysis was performed using Ingenuity Pathway Analysis ™ (IPA). IPA of all proteins with significantly altered levels in the AV group reveals that the majority of these proteins form a complex network. Proteins identified to be regulated in the proteomic screen are given in grey while proteins or protein groups/complexes essential for the network but not found in the screen appear in white. The protein names within the network are provided in the table underneath the network. UniProt/SwissProt accession is given for proteins that have been experimentally identified as regulated. Interestingly, IPA identifies insulin signalling as an important upstream event for the regulation of many relations within the network.

Figure S8 Analysis of regulated protein networks after aversive learning - comparison of protein regulation in striatum (STR), hippocampus (HIP), frontal cortex (FC), auditory cortex (AC). Proteins found to be down-regulated are indicated in blue. Time points after training (6 h and 24h) are pooled. The protein names are as given in the legend to Figure S7.

Figure S9 Analysis of regulated protein networks after aversive learning - comparison of protein regulation in the various brain regions at 6 h or 24 h after aversive learning. STR - striatum, HIP - hippocampus, FC - frontal cortex, AC - auditory cortex, down-regulated proteins are indicated in blue. The protein names are as given in the legend to Figure S7.

Table S1 MIAPE-compliant MS description

Table S2 MIAPE-compliant MSI-description **Table S3** Mean protein abundances in active groups relative to naïve animal groups.
