# Supplementary figures and images for: Synaptic proteome changes in mouse brain regions upon auditory discrimination learning
Source: Proteomics. 2012 Aug 20;12(15-16):2433–44. doi: 10.1002/pmic.201100669 (PMC3509369; doi:10.1002/pmic.201100669)

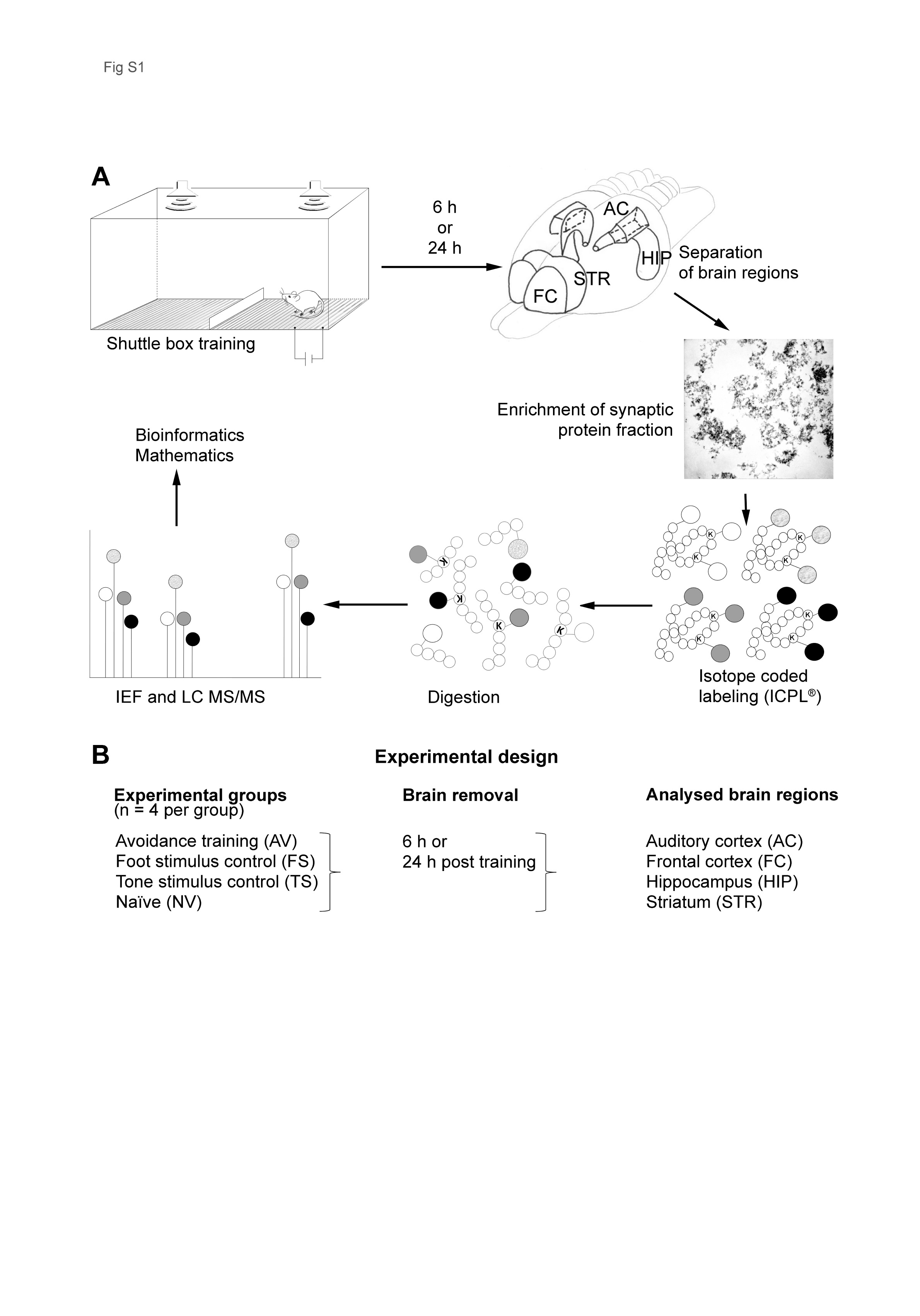

Supplement: Figure S1 — Experimental strategy. (A) Workflow. Mice were trained in a shuttle box to discriminate between rising and falling FM tones. Six or 24 h after avoidance training, mice were sacrificed and striatum (STR), hippocampus (HIP), frontal cortex (FC), and auditory cortex (AC) were removed. Synaptic proteins (PSD-enriched fraction) were collected for each brain region and animal separately. Proteins were labelled by means of ICPL-quadruplex labelling technology and digested with trypsin or GluC endopeptidases. Resulting peptide mixtures were separated by IEF followed by nano-reversed phase HPLC. MS/MS-data sets of four independent replicates were compiled in a database, searched with the Mascot algorithm, and protein abundances relative to the corresponding data from NV mice were calculated. (B) Experimental design of the study. Four male mice (littermates) were either trained or subjected to one of the control treatments and killed after 6 h or 24 h. [file pmic0012-2433-SD1.jpg]

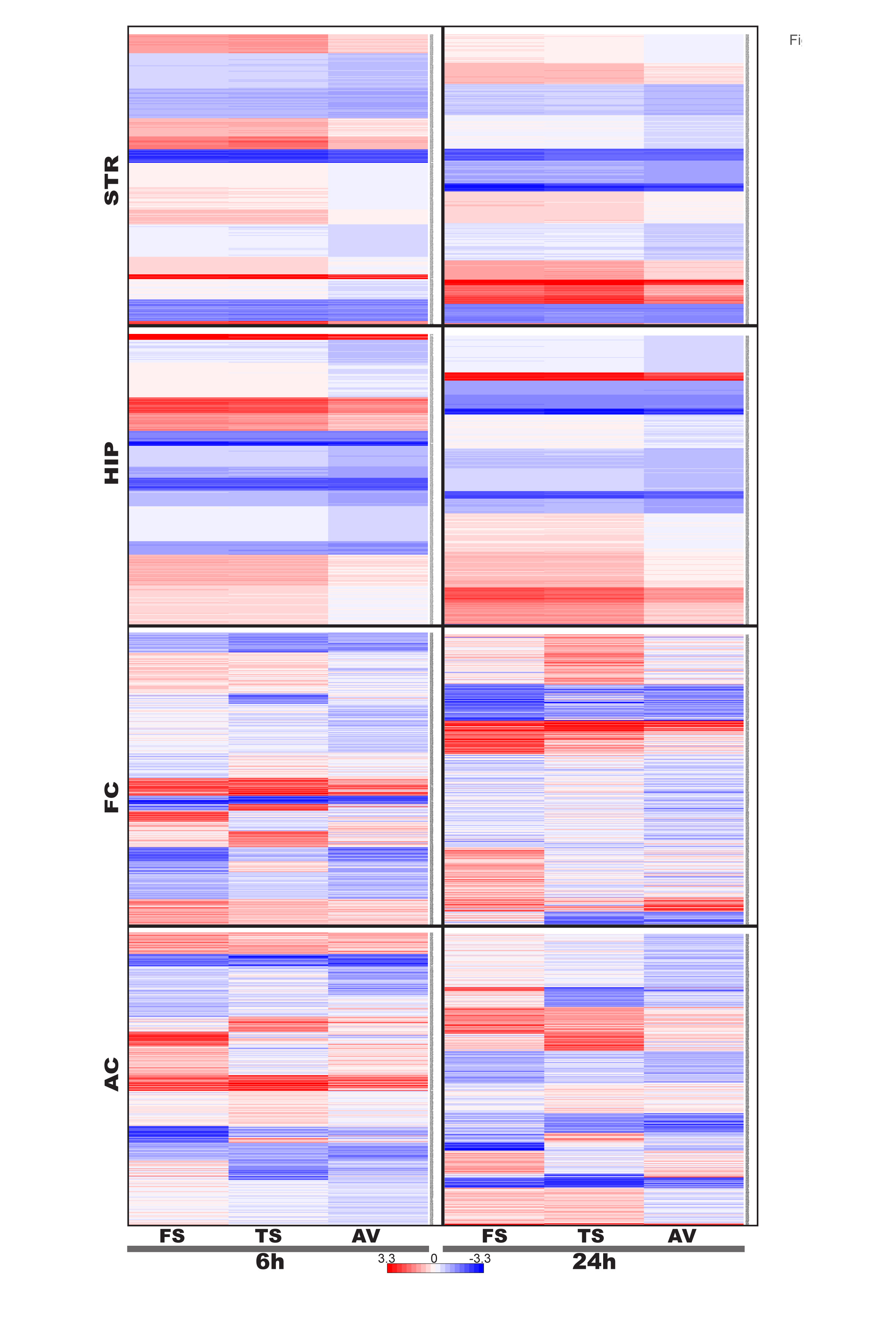

Supplement: Figure S2 — Cluster analysis was performed using the mathematical software package "DanteR" (Pacific Northwest National Laboratory/http://omics.pnl.gov). Log2 of mean values of relative protein levels (FS/NV, TS/NV and AV/NV) were clustered using the K-means algorithm (K=15) on Euclidean distance metrics without data scaling. Protein accession numbers (Swissprot/Uniprot) are printed on right side of each cluster map. They are readable after zooming in the online publication (http://www.SynProt.de/Auditory_discrimination_learning/). In contrast to most other analyses, where proteins with a similar abundance value as in the NV animal (from factor 0.9 to 1/0.9) were excluded, cluster analysis included all identified quantifiable proteins. [file pmic0012-2433-SD2.jpg]

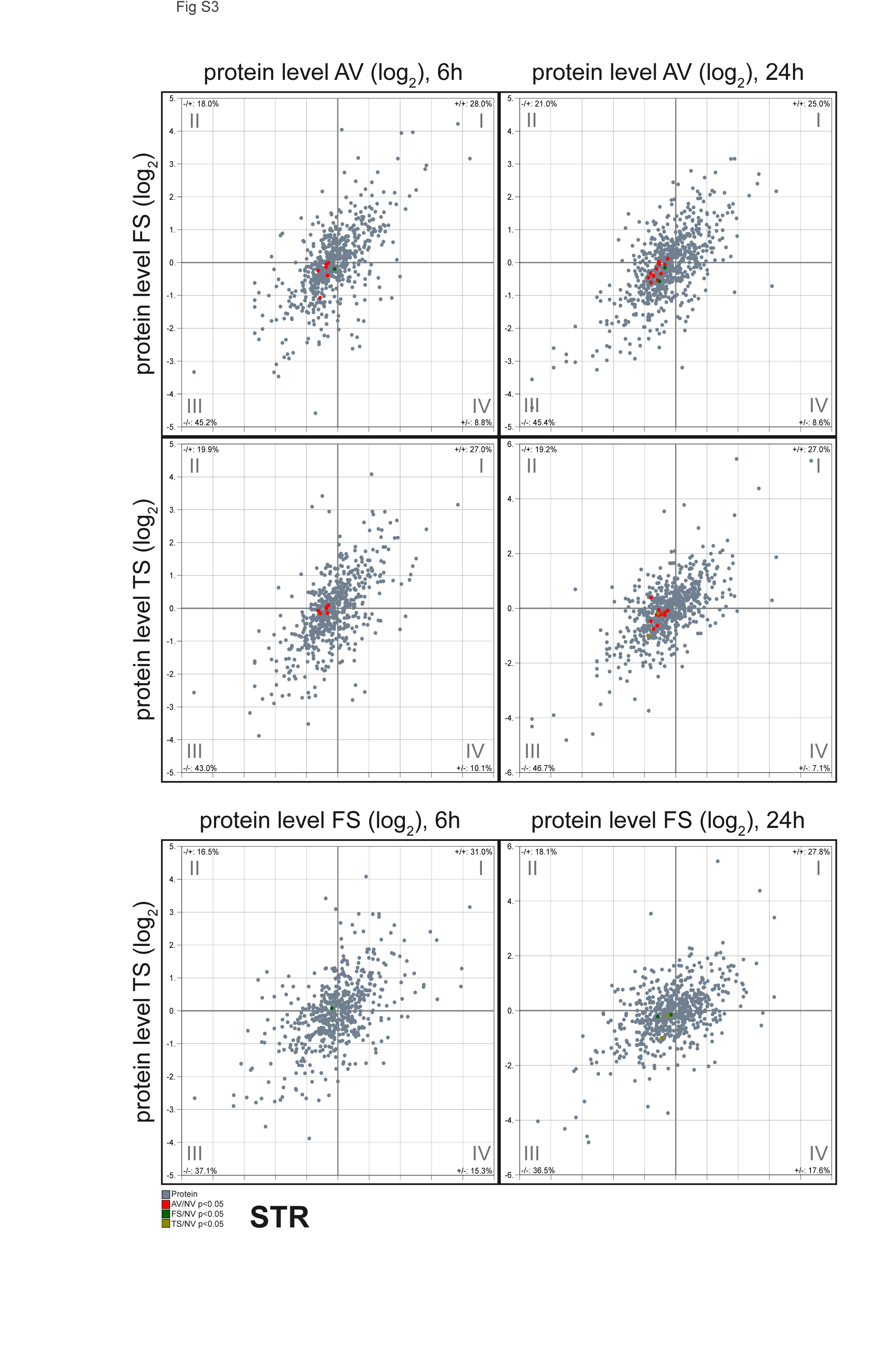

Supplement: Figure S3 — Correlation plots of relative synaptic levels of striatal proteins. Mean abundances relative to NV of striatal proteins monitored 6 h (left) and 24 h (right) after behavioural experiments are plotted on a double logarithmic scale, comparing AV and FS (upper part), AV and TS (middle part), and FS and TS (lower part). Each data point represents a unique protein. Spot colours other than gray correspond to those used in Table S3 (Supporting Information). The percentage of proteins present in each quadrant is given. Proteins with abundance values similar to the NV group (0.9 - 1/0.9) are excluded from plotting and calculation. Corresponding Swissprot/UniProt database accession numbers are available in the interactive plots, which will be available on http://www.synprot.de/Auditory_discrimination_learning/). [file pmic0012-2433-SD3.jpg]

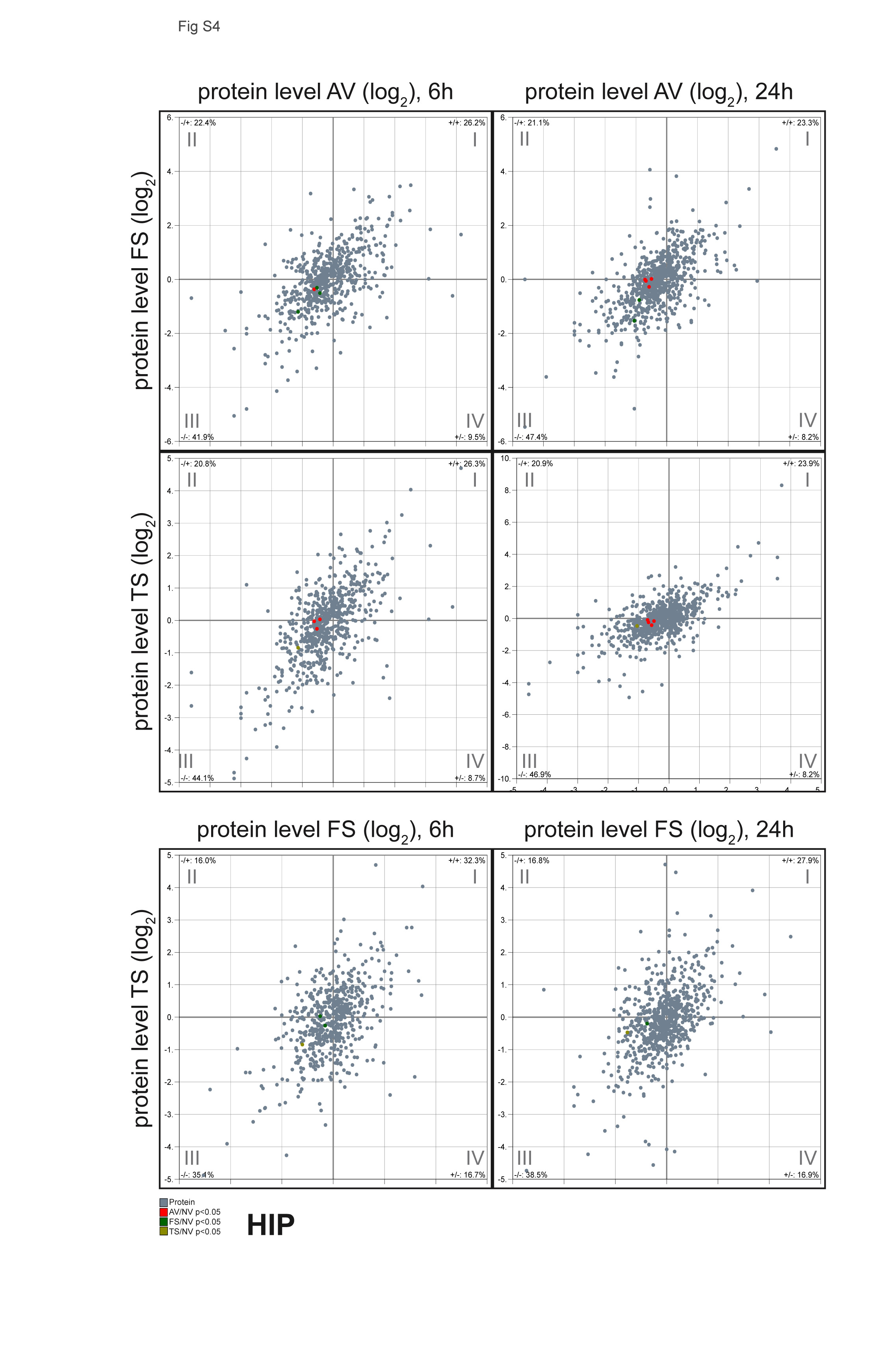

Supplement: Figure S4 — Correlation plots of relative synaptic levels of hippocampal proteins. Mean abundances relative to NV of auditory cortex proteins monitored 6 h (left) and 24 h (right) after behavioural experiments are plotted on a double logarithmic scale, comparing AV and FS (upper part), AV and TS (middle part), and FS and TS (lower part). Each data point represents a unique protein. Spot colours other than gray correspond to those used in Table S3 (Supporting Information). The percentage of proteins present in each quadrant is given. Proteins with abundance values similar to the NV group (0.9 - 1/0.9) are excluded from plotting and calculation. Corresponding Swissprot/UniProt database accession numbers are available in the interactive plots, which will be available on http://www.synprot.de/Auditory_discrimination_learning/). [file pmic0012-2433-SD4.jpg]

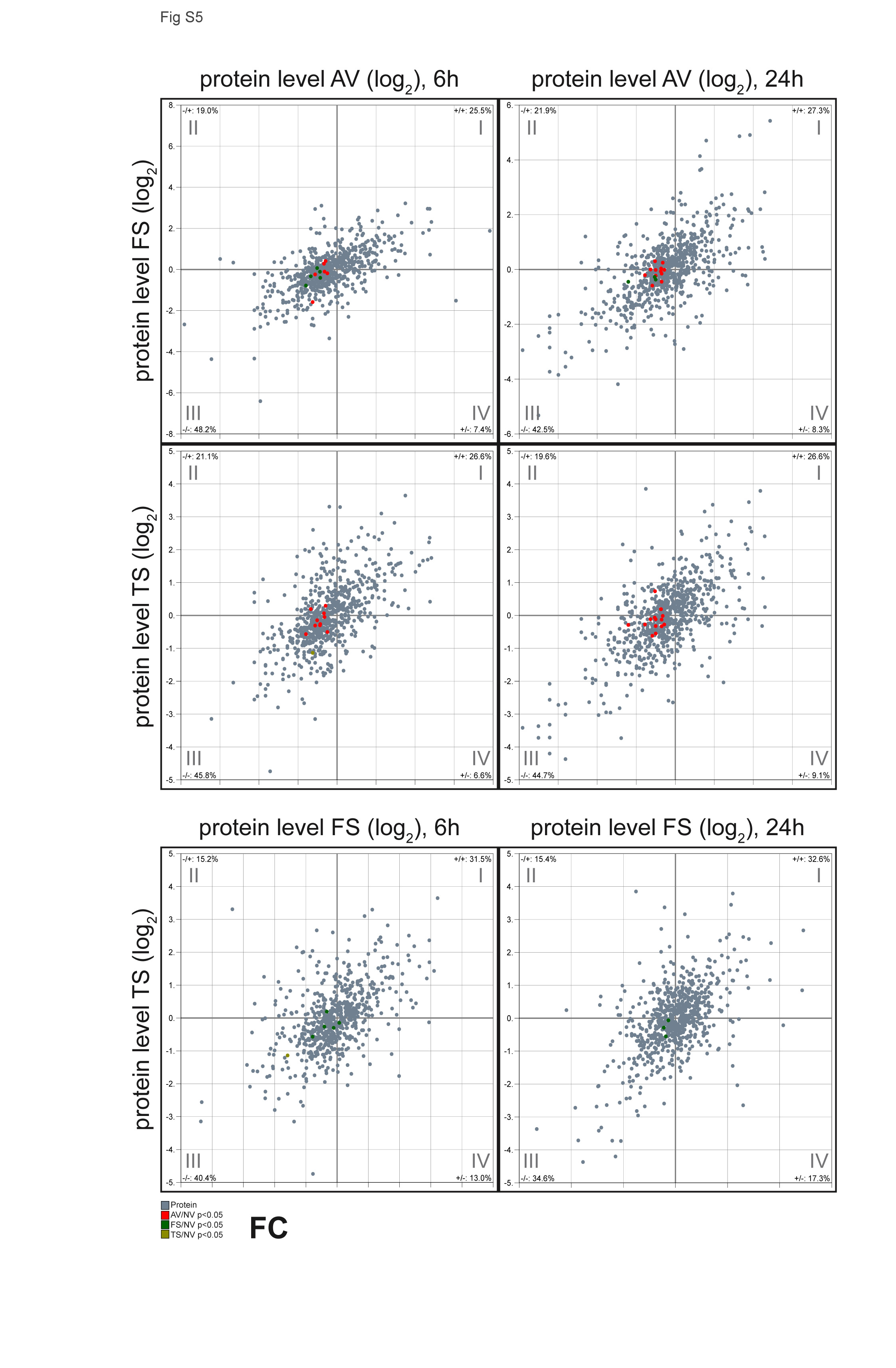

Supplement: Figure S5 — Correlation plots of relative synaptic levels of frontal cortex proteins. Mean abundances relative to NV of frontal cortex proteins monitored 6 h (left) and 24 h (right) after behavioural experiments are plotted on a double logarithmic scale, comparing AV and FS (upper part), AV and TS (middle part), and FS and TS (lower part). Each data point represents a unique protein. Spot colours other than gray correspond to those used in Table S3 (Supporting Information). The percentage of proteins present in each quadrant is given. Proteins with abundance values similar to the NV group (0.9 - 1/0.9) are excluded from plotting and calculation. Corresponding Swissprot/UniProt database accession numbers are available in the interactive plots, which will be available on http://www.synprot.de/Auditory_discrimination_learning/). [file pmic0012-2433-SD5.jpg]

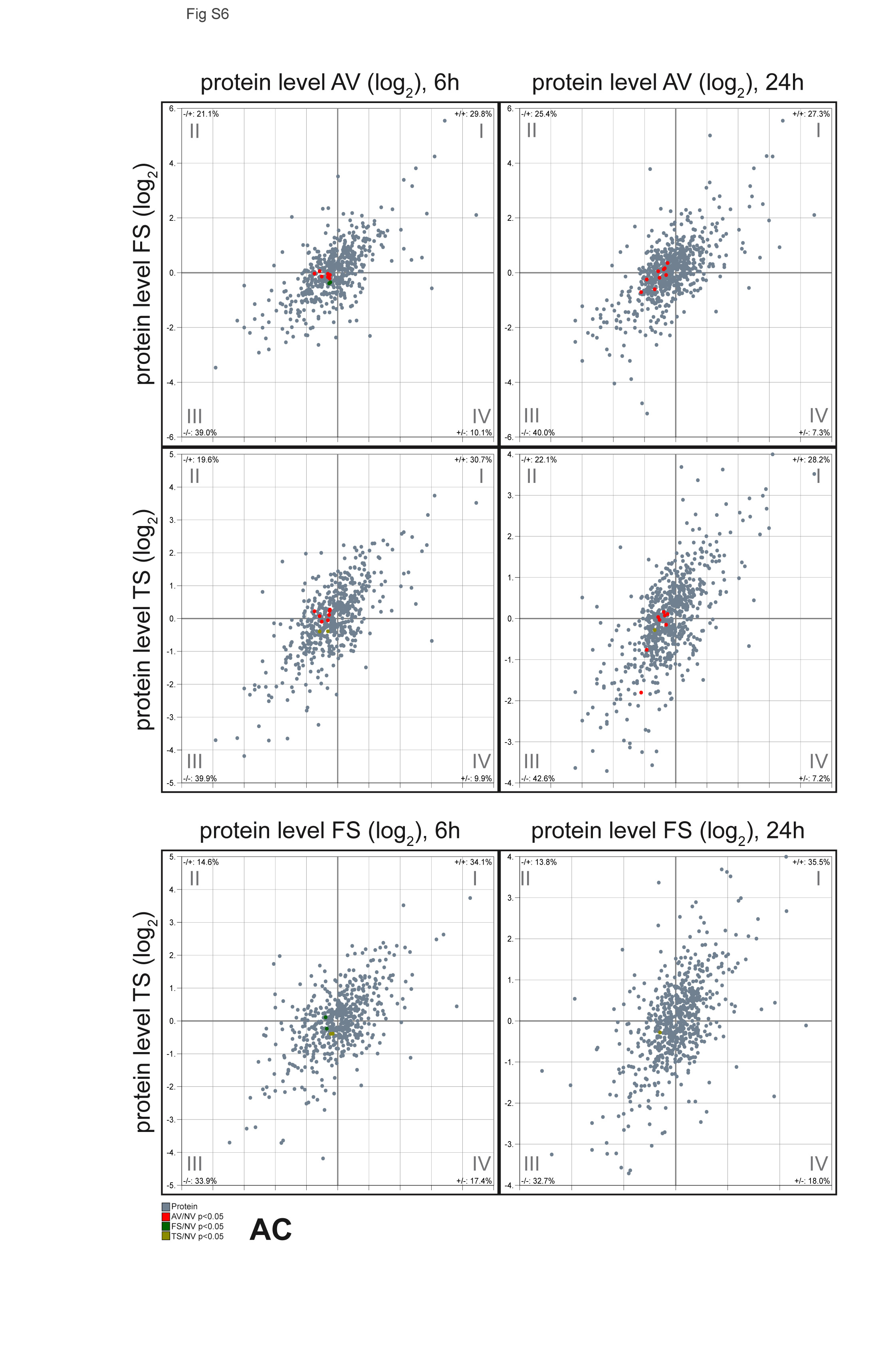

Supplement: Figure S6 — Correlation plots of relative synaptic levels of auditory cortex proteins. Mean abundances relative to NV of auditory cortex proteins monitored 6 h (left) and 24 h (right) after behavioural experiments are plotted on a double logarithmic scale, comparing AV and FS (upper part), AV and TS (middle part), and FS and TS (lower part). Each data point represents a unique protein. Spot colours other than gray correspond to those used in Table S3 (Supporting Information). The percentage of proteins present in each quadrant is given. Proteins with abundance values similar to the NV group (0.9 - 1/0.9) are excluded from plotting and calculation. Corresponding Swissprot/UniProt database accession numbers are available in the interactive plots, which will be available on http://www.synprot.de/Auditory_discrimination_learning/). [file pmic0012-2433-SD6.jpg]

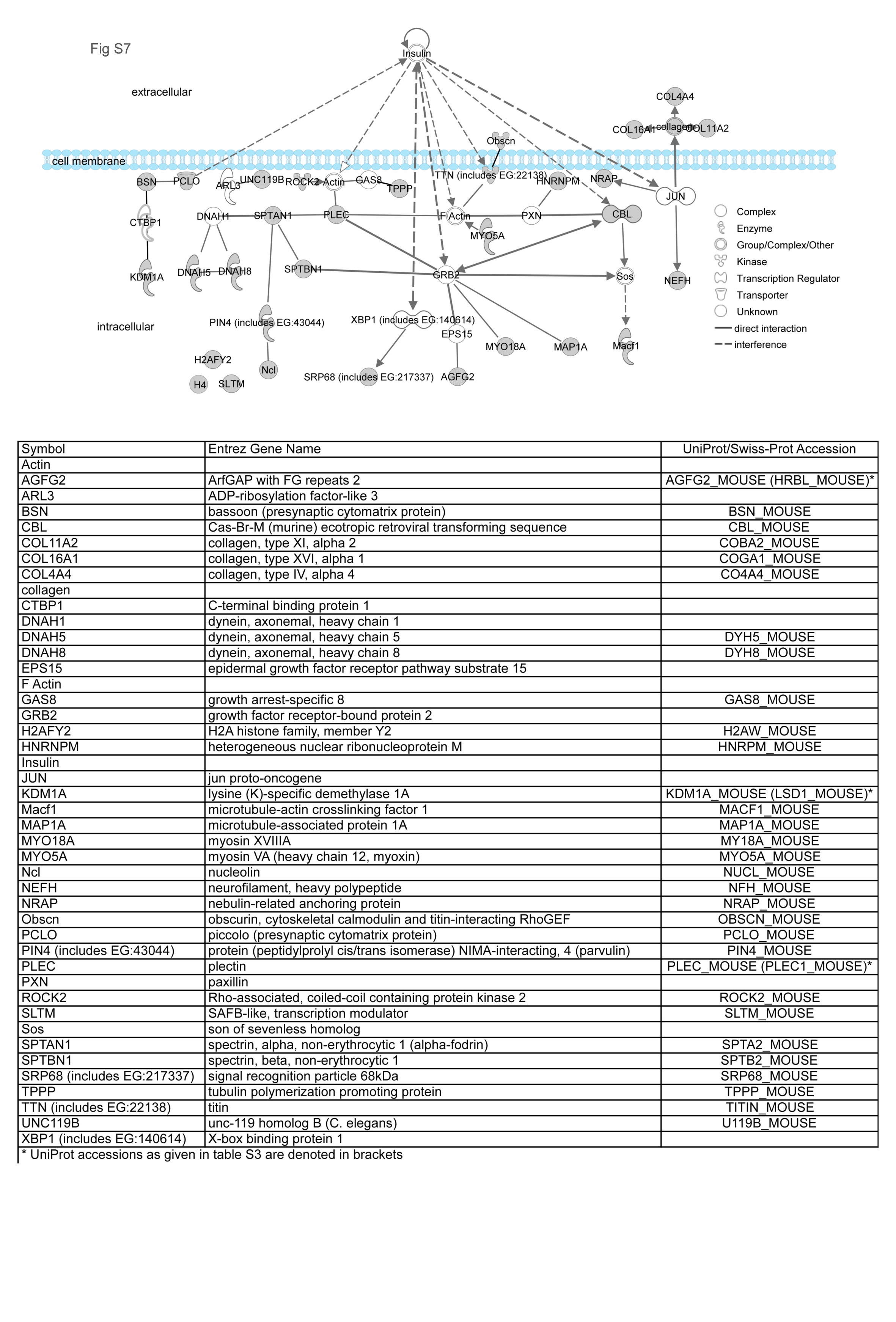

Supplement: Figure S7 — Analysis of regulated protein networks after aversive learning-summary of all brain areas and time points. Network analysis was performed using Ingenuity Pathway Analysis ™ (IPA). IPA of all proteins with significantly altered levels in the AV group reveals that the majority of these proteins form a complex network. Proteins identified to be regulated in the proteomic screen are given in grey while proteins or protein groups/complexes essential for the network but not found in the screen appear in white. The protein names within the network are provided in the table underneath the network. UniProt/SwissProt accession is given for proteins that have been experimentally identified as regulated. Interestingly, IPA identifies insulin signalling as an important upstream event for the regulation of many relations within the network. [file pmic0012-2433-SD7.jpg]

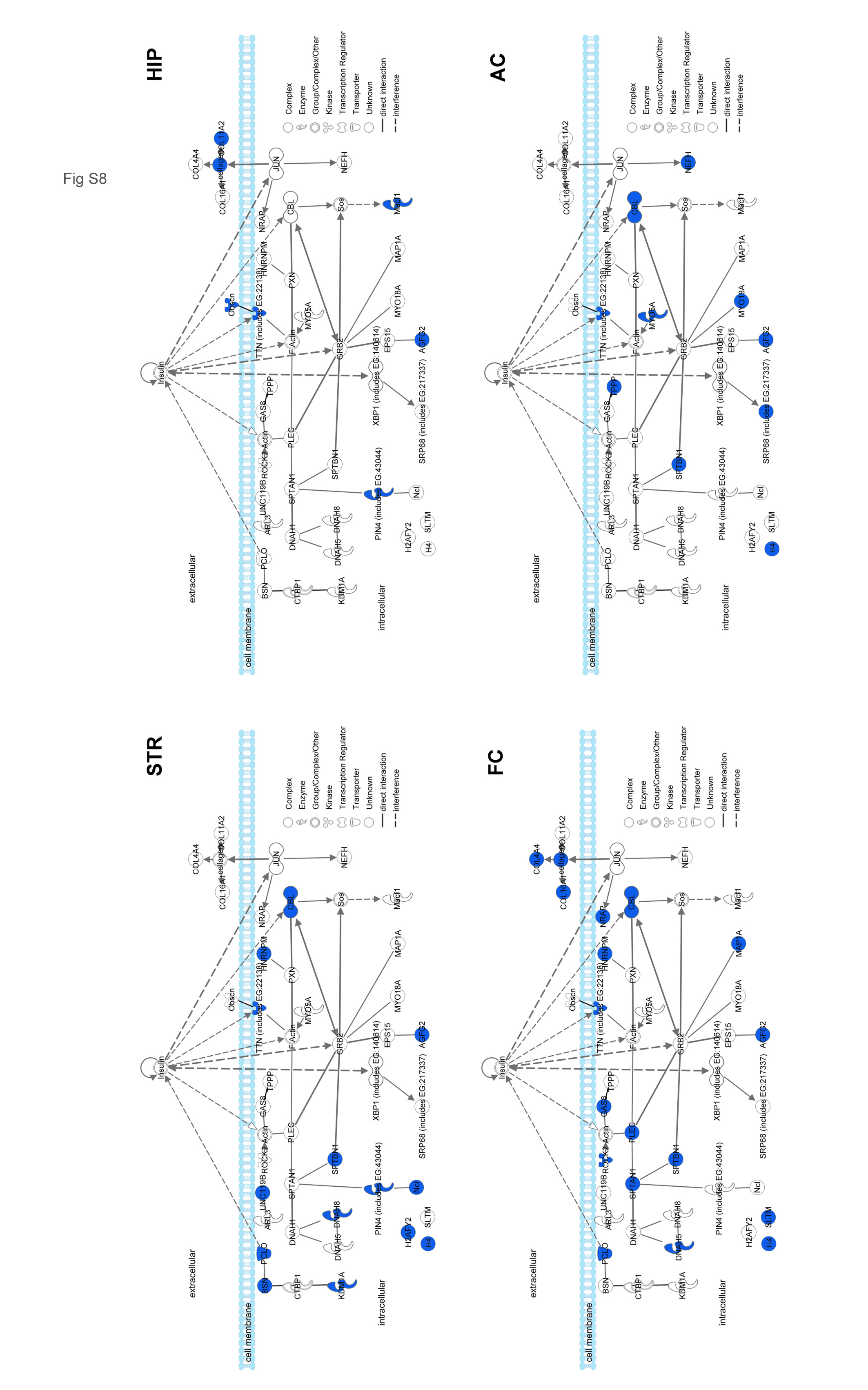

Supplement: Figure S8 — Analysis of regulated protein networks after aversive learning - comparison of protein regulation in striatum (STR), hippocampus (HIP), frontal cortex (FC), auditory cortex (AC). Proteins found to be down-regulated are indicated in blue. Time points after training (6 h and 24h) are pooled. The protein names are as given in the legend to Figure S7. [file pmic0012-2433-SD8.jpg]

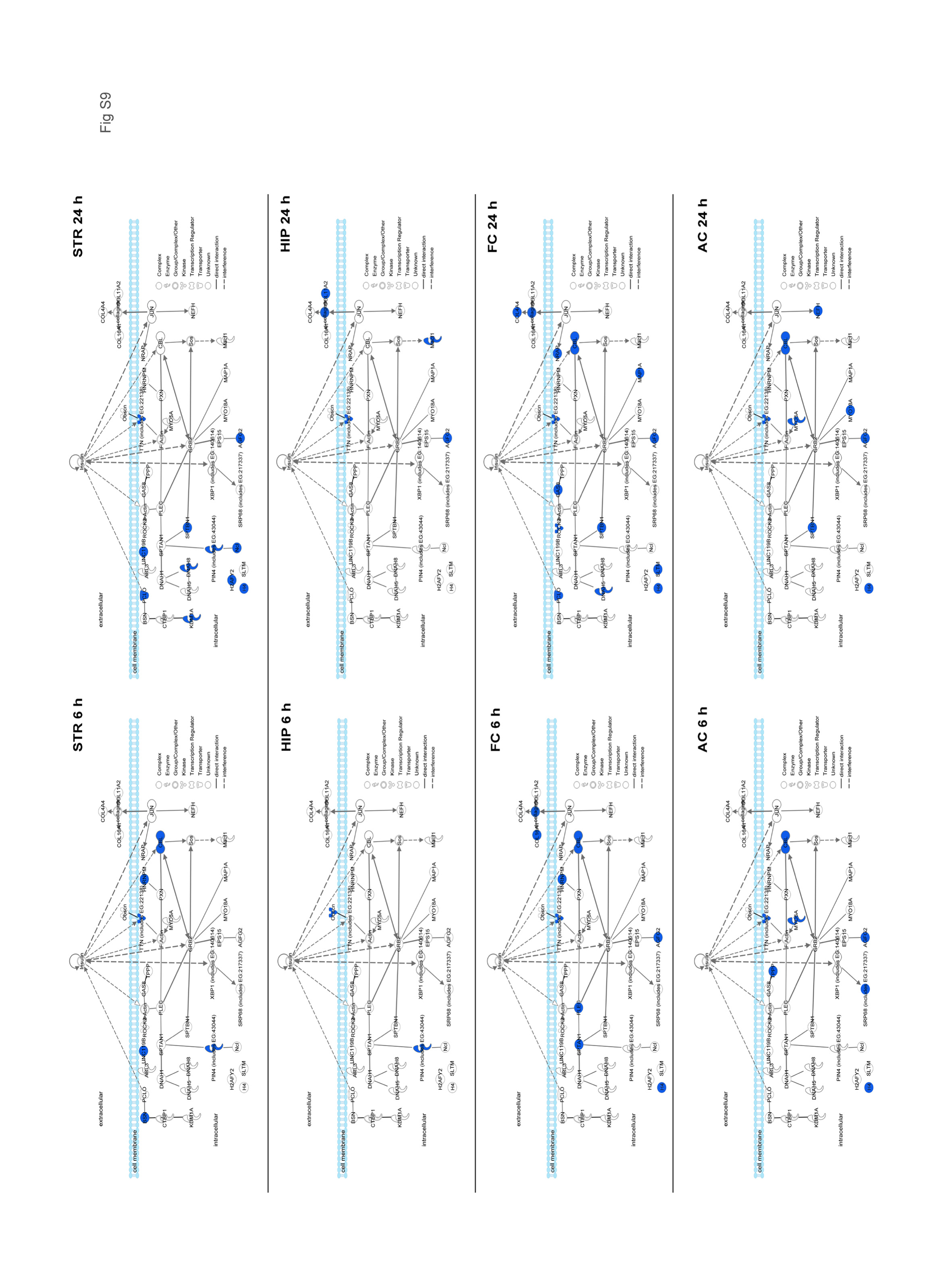

Supplement: Figure S9 — Analysis of regulated protein networks after aversive learning - comparison of protein regulation in the various brain regions at 6 h or 24 h after aversive learning. STR - striatum, HIP - hippocampus, FC - frontal cortex, AC - auditory cortex, down-regulated proteins are indicated in blue. The protein names are as given in the legend to Figure S7. [file pmic0012-2433-SD9.jpg]
