# Supplementary material for: Synaptic proteome changes in mouse brain regions upon auditory discrimination learning
Source: Proteomics. 2012 Aug 20;12(15-16):2433–44. doi: 10.1002/pmic.201100669 (PMC3509369; doi:10.1002/pmic.201100669)
Supplement: Table S1 — MIAPE-compliant MS description [file pmic0012-2433-SD10.doc]

| **MIAPE-compliant MS-description:** | |
| --- | --- |
| **Classification** | **Definition** |
| ***1. General features — (a) Global descriptors*** |  |
| Responsible person | T. Kähne, PhD, Institute of Exptl. Internal Medicine, University Magdeburg, Leipziger Str. 44, 39120 Magdeburg, Germany |
| Instrument manufacturer, model | Bruker Daltonics, Germany, HCT ultra, PTM Discovery System ETDII, Iontrap-Mass Spectrometer |
| Customisations | no customisations |
| ***1. General features — (b) Control and analysis software*** |  |
| Software name and version | Esquire Control 6.2, HyStar 3.2 SR2 |
| Switching criteria (tandem only) | Precursor selection threshold: 3000 Abs  Preferred charge state: double  SILE triggered delta mass: 1.97, 3.99, 4.05, 5.96, 6.02, 10.01  SILE selection: within top 100 (tolerance 0.2 m/z)  SILE selection: heavy/light >0.1 and < 10.0  MS/MS selection: largest peak only  Five precursor ions per MS scan  Active exclusion after 2 spectra, release after 1 min |
| Isolation width (global, or by MS level) | Isolation with: 4.0 m/z |
| Location of ‘parameters’ file | not supplied |
| ***2. Ion sources — (a) Electrospray Ionisation (ESI)*** |  |
| Supply type (static, or fed) | Fed by nano-HPLC (U3000, Dionex, Germany) |
| Interface manufacturer, model | Orthogonal nano-online spray source, Bruker Daltonics, Germany |
| Sprayer type, coating, manufacturer, | Coated PicoTip Emitters (FS360-20-10-D), New Objective, USA |
| Relevant voltages where appropriate (tip, cone, acceleration) | Positive mode  Emitter-voltage: 1200 V  Capillary current: max. 100nA |
| Whether in-source dissociation performed | no |
| Other parameters if discriminant for the experiment | Passive spray, without nebulising gas  Dry gas: 160°C, 10l/min nitrogen |
| ***3. Post-source component — (c) Ion rap*** |  |
| Final MS stage achieved | MS^2 (CID and ETD, respectively) |
| Gas type and pressure (bar) | Paul trap  Helium, 10^-6^ bar |
| Collision energy | Smart fragmentation (start ampl 30.0% - end ampl 300%)  Frequency: 781/3 kHz  Fragmentation time: 40ms  Low CID cutoff: 17% |
| Electron transfer based fragmentation ETD | Auto MS^2 after CID  Max. ETD precursor mass: 1500 m/z  CutOff 170 m/z  Fragmentation time: 100 ms  Smart Decomp CID/ETD Ampl 0.5 V |

| ***3. Post-source component — (f) Detectors*** |  |
| --- | --- |
| Detector | SEV, 1730 V |
| ***4. Spectrum and peak list generation and annotation — (a) Spectrum description*** |  |
| Location of source (‘raw’) file including file  name and type | All raw-data files are locally stored and backuped on HP blade server cluster  Processed acquisition data were stored in a ProteinScape 3.0 SQL-based Server, no public availability |
| MS level for this spectrum | Raw data files consist of MS and MS^2 spectra |
| Ion mode for this spectrum | Always positive mode |
| Precursor m/z and charge, with the full mass spectrum containing that peak (for MS level 2 and higher) | For all MS^2 spectra: precursor m/z and fragmentation mode (CID or ETD) is provided as a part of the raw data file name |
| ***4. Spectrum and peak list generation and annotation — (b) Peak list generation*** |  |
| Used software | DataAnalysis 4.0 SP2, Bruker Daltonics, Germany |
| Smoothing; whether applied, parameters | Sawitzky Golay, with 7.263 s, 1 cycle |
| Background threshold, or algorithm used | Intensity threshold: 10.000 |
| Signal-to-noise estimation and method | S/N threshold: 5  Correlation coefficient threshold: 0.7  Minimum compound length: 10 spectra |
| Percentage peak height for centroiding | Quality factor threshold: 0.9  S/N threshold: 2  Relative intensity threshold (base peak): 0%  Absolute intensity threshold: 100  Maximum charge state: 4 |
| Whether charge states were calculated, spectra were deconvoluted | Deconvolution was performed for MS full scan (abundance cutoff 2%) and MS^2 (abundance cutoff 0.5%)  Fragments qualified by Amino acids |
| ***4. Peak list generation and annotation — (c) Quantitation for selected ions (in addition to 4a) and 4b)*** |  |
| Experimental protocol, canonical reference  where available with deviations | ProteinScape 3.0, WarpLC 1.3 |
| Number of combined samples and MS runs analysed | 40 LC-MS/MS runs have been combined for each quantitation run |
| Quantitation approach (e.g. integration) | Calculation by peak intensity  Mass tolerance: 0.2 Da  Retention time tolerance: 70 s  Accepted charge states: 4  Accepted regulation: 0.01<x/y< 100 |
| Normalisation technique | not applied |
| Location of quantitation data | Quantitation data were stored in a ProteinScape 3.0 SQL-based Server, no public availability |

| **MIAPE-compliant MSI-description** | |
| --- | --- |
| **Classification** | **Definition** |
| ***1. General features — (a) Global descriptors*** |  |
| Responsible person | T. Kähne, PhD, Institute of Exptl. Internal Medicine, University Magdeburg, Leipziger Str. 44, 39120 Magdeburg, Germany |
| Software name, version and manufacturer | ProteinScape 3.0, Bruker Daltonics, Germany, Mascot 2.1, Matrix Science, UK |
| Customisations | no customisations |
| Availability of the software | Local MASCOT Server, 4 processor licence |
| Location of the files generated | Files were stored in a ProteinScape 3.0 SQL-based Server and on local Mascot server, no public availability |
| ***2. Input data and parameters – (a) input data*** |  |
| Description and type of MS data | Original format: Bruker.yep  Extracted data for database search: xml-format |
| Availability of MS data | Locally stored (ProteinScape 3.0 SQL-based Server), no public availability |
| ***2. Input data and parameters – (b) input parameters*** |  |
| Database queried | Swissprot 56.6 |
| Taxonomical restrictions | Mus |
| Description of tool and scoring scheme | ProteinExtractor (ProteinScape, Bruker Daltonics, Germany) |
| Specified cleavage agent(s) | Trypsin, V8-DE, respectively |
| Allowed number of missed cleavages | 1 |
| Additional parameters related to cleavage | no |
| Permissible amino acids modifications | fixed: Cabamidomethyl  variable:   | ICPL (K) | | --- | | ICPL (Protein N-term) | | ICPL:13C(6) (K) | | ICPL:13C(6) (Protein N-term) | | ICPL:13C(6)2H(4) (K) | | ICPL:13C(6)2H(4) (Protein N-term) | | ICPL:2H(4) (K) | | ICPL:2H(4) (Protein N-term) | | Oxidation (M) | |
| Precursor-ion and fragment-ion mass tolerance for tandem MS | Precursor ion tolerance: 1.4 Da  Fragment ion tolerance: 0.5 Da |
| Thresholds; minimum scores for peptides,proteins | Peptide score threshold: 20  Protein score threshold: 80  Min. 1 peptide(s) with score > 40 |
| ***3. The output from the procedure – (c) quantitation for selected ions*** |  |
| Quantitation approach (e.g. 4plex-iTRAQ, ICAT, cICAT, COFRADIC) | ICPL quadruplex, Serva, Germany |
| Quantity measurement | Calculation by peak intensity |
| Data transformation and normalization technique | WarpLC 1.3, Bruker Daltonics, Germany  Mass tolerance: 0.2 Da  Retention time tolerance: 70 s  Accepted charge states: 4  Accepted regulation: 0.1<x/y< 10 |
| Number of replicates (biological and technical) | 4 |
| Acceptance criteria (including measure of errors) | Acceptance of a quantified protein by at least 3 different quantifiable peptides (peptide score >20) |
| Results from controls (when described) | All protein levels have been calculated relative to naïve controls (level set to 1) |
| ***4. Interpretation and validation*** |  |
| Assessment and confidence given to the identification and quantitation (description of methods, thresholds, values, etc.) | Mean protein abundances in PSD-enriched fractions derived from trained mice (AV), foot-shock controls (FS), and tone controls (TC) have been normalised to the corresponding values from naive mice (NV) (3≤n≤4 per group) |
| Results of statistical analysis | One sample t-test, hypothesized mean = 1  Heatmap cluster analysis: “DanteR” (*Pacific Northwest National Laboratory (PNNL) and http://*OMICS.PNL.GOV  Cluster settings: K-means clustering method (K set to 15) on Euclidean distance metric |
| Inclusion/exclusion of the output of the software are provided (description of what part of the output has been kept, what part has been rejected) | Inclusions for Correlation plotting and Cluster analysis: Peptide mascot score > 20, Protein mascot score >80 (min. 1 peptide(s) with mascot score > 40), Assessment done by ProteinExtractor (software module of ProteinScape 3.0, Bruker Daltonics, Germany)  Inclusions for table 2 and figure 4: protein mean ratio p<0.05, significant difference from 1 and 0.05<p<0.1, marginally significant difference from 1   |  | | --- | |  | |
